# Water temperature drives phytoplankton blooms in coastal waters

**DOI:** 10.1371/journal.pone.0214933

**Published:** 2019-04-05

**Authors:** Thomas Trombetta, Francesca Vidussi, Sébastien Mas, David Parin, Monique Simier, Behzad Mostajir

**Affiliations:** 1 MARBEC (Marine Biodiversity, Exploitation and Conservation), Centre National de la Recherche Scientifique, Université de Montpellier, Institut Français de Recherche pour l’Exploitation de la Mer, Institut de Recherche pour le Développement, Montpellier, France; 2 MEDIMEER (Mediterranean Platform for Marine Ecosystems Experimental Research), Observatoire de Recherche Méditerranéen de l’Environnement, Centre National de la Recherche Scientifique, Université de Montpellier, Institut de Recherche pour le Développement, Institut National de Recherche en Sciences et Technologies pour l’Environnement et l’Agriculture, Sète, France; 3 MARBEC (Marine Biodiversity, Exploitation and Conservation), Institut de Recherche pour le Développement, Centre National de la Recherche Scientifique, Université de Montpellier, Institut Français de Recherche pour l’Exploitation de la Mer, Sète, France; Stazione Zoologica Anton Dohrn, ITALY

## Abstract

Phytoplankton blooms are an important, widespread phenomenon in open oceans, coastal waters and freshwaters, supporting food webs and essential ecosystem services. Blooms are even more important in exploited coastal waters for maintaining high resource production. However, the environmental factors driving blooms in shallow productive coastal waters are still unclear, making it difficult to assess how environmental fluctuations influence bloom phenology and productivity. To gain insights into bloom phenology, Chl *a* fluorescence and meteorological and hydrological parameters were monitored at high-frequency (15 min) and nutrient concentrations and phytoplankton abundance and diversity, were monitored weekly in a typical Mediterranean shallow coastal system (Thau Lagoon). This study was carried out from winter to late spring in two successive years with different climatic conditions: 2014/2015 was typical, but the winter of 2015/2016 was the warmest on record. Rising water temperature was the main driver of phytoplankton blooms. However, blooms were sometimes correlated with winds and sometimes correlated with salinity, suggesting nutrients were supplied by water transport via winds, saltier seawater intake, rain and water flow events. This finding indicates the joint role of these factors in determining the success of phytoplankton blooms. Furthermore, interannual variability showed that winter water temperature was higher in 2016 than in 2015, resulting in lower phytoplankton biomass accumulation in the following spring. Moreover, the phytoplankton abundances and diversity also changed: cyanobacteria (< 1 μm), picoeukaryotes (< 1 μm) and nanoeukaryotes (3–6 μm) increased to the detriment of larger phytoplankton such as diatoms. Water temperature is a key factor affecting phytoplankton bloom dynamics in shallow productive coastal waters and could become crucial with future global warming by modifying bloom phenology and changing phytoplankton community structure, in turn affecting the entire food web and ecosystem services.

## Introduction

Ocean phytoplankton generate almost half of global primary production [1], making it one of the supporting pillars of marine ecosystems, controlling both diversity and functioning. Phytoplankton in temperate and subpolar regions are characterized by spring blooms, a seasonal phenomenon with rapid phytoplankton biomass accumulation due to a high net phytoplankton growth rate [2]. This peak biomass of primary producers in the spring supports the marine food web through carbon transfer to higher trophic levels from zooplankton to fishes. Spring phytoplankton blooms are a common phenomenon in all aquatic systems, from open oceans to coastal waters and from transient waters to inland freshwaters. The magnitude, timing and duration of blooms are as diverse as the ecosystems in which they occur.

For more than half a century, several paradigms and theories have been developed to explain the general mechanism of bloom initiation; however from the earliest critical depth hypothesis of Sverdrup in 1953 [3] they have been, and still are, subject to scientific debate. The critical depth hypothesis is a bottom-up model based on abiotic drivers and proposes that a bloom starts when there is sufficient solar radiation and the surface mixing layer becomes shallower. This change induces stratification [4–7], allowing phytoplankton to remain in the surface layer, such that their growth rates overcome their losses (i.e., mostly by zooplankton grazing). This hypothesis has been questioned several times, as observations have shown that blooms can occur in the absence of stratification. The mixing layer is defined by density, and since the critical depth hypothesis was put forth, other bottom-up mixing models have been proposed to explain bloom onset. These models are based on actively mixed layers that may occur in turbulence windows (the critical turbulence hypothesis [8]) or under deep convection shutdown (the convection shutdown hypothesis [7]), allowing the phytoplankton to remain in the surface layer long enough to benefit from favorable light conditions and start a bloom.

Recently, the disturbance recovery hypothesis of Behrenfeld et al. [9,10] formalized a new theory based on biotic drivers (top-down control) that had already been suggested several years before [11,12]. This biotic driver theory proposes that a disturbance factor, such as environmental forcing, disrupts zooplankton-phytoplankton predator-prey interactions, allowing the prey (phytoplankton) to grow rapidly, creating a bloom. Later, when the predator-prey interactions recover, the bloom ends as the losses by predation overwhelm the gains in prey biomass. This general ecological theory was proposed for the North Atlantic, where the establishment of deep-water mixing provides the ecosystem disturbance that disrupts predator-prey interactions. In other systems, different sources of disturbance can play this role, such as monsoon forcing in the Arabian Sea [13] or upwelling in coastal systems [11]. However, the role played by bottom-up and top-down drivers in phytoplankton spring blooms is still a source of debate [14,15].

Coastal waters and lakes are highly dynamic and productive ecosystems where phytoplankton blooms are common [16,17]. Coastal phytoplankton blooms are a major ecological event providing a substantial part of the annual primary production and energy transfer supporting the entire marine food web. These highly productive periods in coastal systems occur mainly in spring and autumn and are believed to be influenced by several factors such as increasing irradiance, anticyclones and nutrient inputs [18–20]. However, clear links between general theories and field observations in coastal waters have not yet been established. In particular, the factors that might disturb predator-prey relationships in these shallow systems have been neglected. Coastal waters, including estuaries, sea grass beds, coral reefs and continental shelves, cover only 6% of the world’s surface but provide between 22% and 43% of the estimated value of the world’s ecosystem services [21]. In addition to hosting major biochemical and ecological processes, such as nutrient cycling and biological control, and providing habitats and refugia, coastal waters are of great economic importance for local populations because they provide food, raw materials and recreational activities [22]. Coastal waters can be strongly affected by climatic events due to their higher reactivity and lower inertia compared to open-ocean waters, making them highly sensitive to environmental forcing fluctuations [23].

Nevertheless, in open oceans, increasing water temperature due to global warming changes the start and end timing of the blooms, and reduces their amplitude, affecting the survival and hatching time of commercially important species [24,25]. Furthermore, experimental studies have shown that warmer conditions change the composition and trophic interactions of plankton communities, propagating the effects to higher trophic levels [26–28]. However, the impact of environmental forcing factors on spring bloom phenology in shallow waters is not well studied, making it difficult to assess the future of coastal water ecosystems in a global climate change context.

The objective of the present study was to investigate spring bloom dynamics and the associated phytoplankton diversity in a typical shallow coastal system to identify the environmental factors triggering the blooms. The study combined high-frequency monitoring of chlorophyll *a* (Chl *a*) fluorescence as a proxy of phytoplankton biomass, high-frequency monitoring of environmental parameters in the air and water and weekly water sampling of the phytoplankton community and nutrients in the water. The study was undertaken during winter and spring of 2015 and 2016 in Thau Lagoon, a typical productive coastal site on the edge of the Mediterranean Sea.

## Materials and methods

### Study site

**Fig 1:**
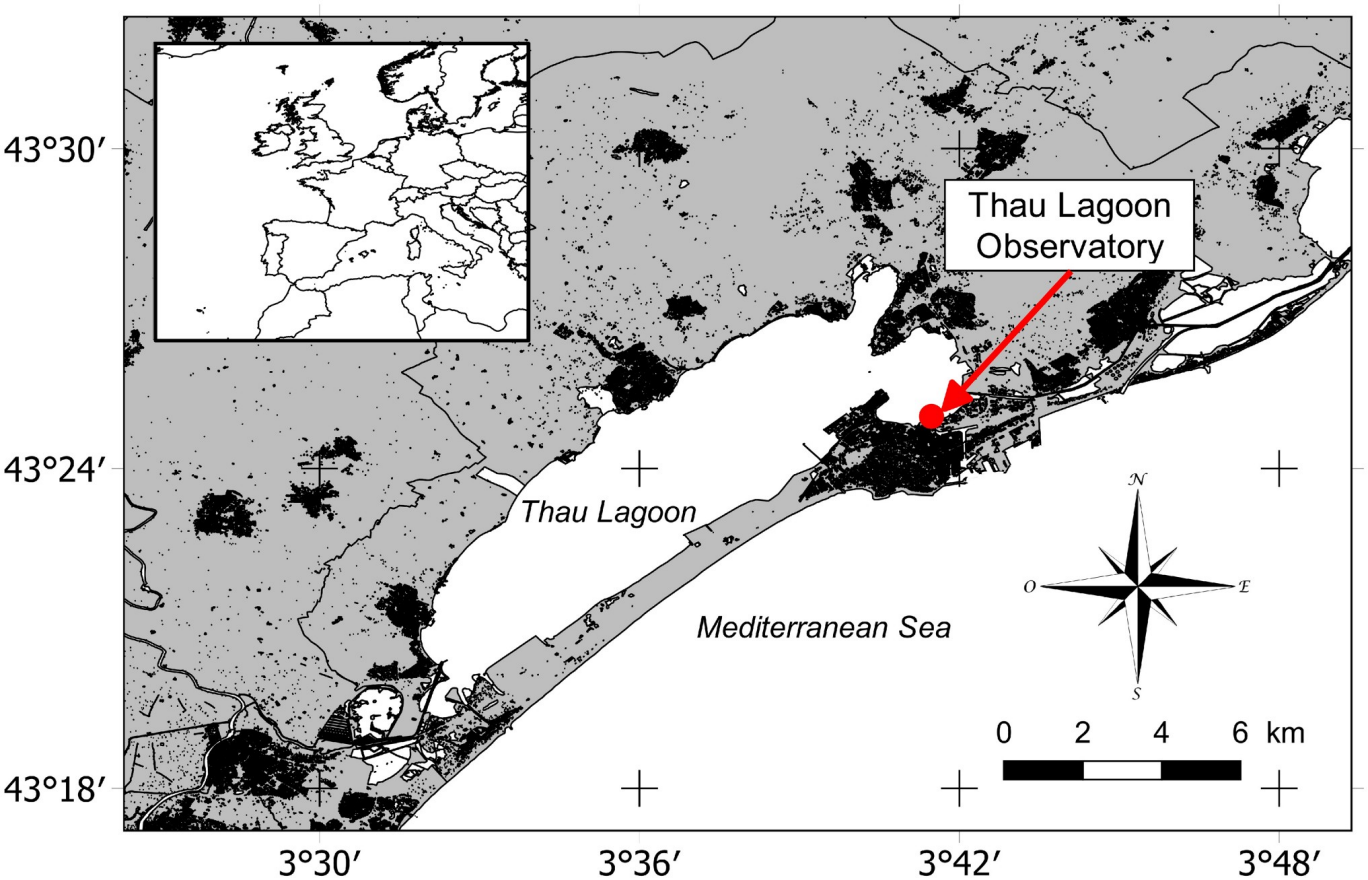
Location of the sampling station.

The study site, Thau Lagoon (Fig 1), was chosen as it is a productive coastal site of economic interest (principally oyster farms representing 10% of French production) characterized by large seasonal temperature variation (from approximately 4 to 30°C throughout the year [29]). Thau Lagoon is located on the French coast of the northwestern Mediterranean Sea (43°24’00” N, 3°36’00” E). It is a shallow lagoon with a mean depth of 4 m, a maximum depth of 10 m (excluding deep depressions) and an area of 75 km^2^ and is connected to the Mediterranean Sea by three channels. Thau Lagoon is a mesotrophic lagoon with a turnover rate of 2% (50 days), mostly through the Sète channel [30]. Recent studies have suggested that the lagoon is a phosphorus- and nitrogen-limited system [31]. The water column was monitored at a high frequency (at mid-depth, approximately 1.5 m below the surface) using several sensors at a fixed station (Coastal Mediterranean Thau Lagoon Observatory 43°24’53” N, 3°41’16” E) [32] near the Mediterranean platform for Marine Ecosystem Experimental Research (MEDIMEER), in the city of Sète. This fixed station was also used for weekly water sampling. The depth of the station is 2.5–3 m, and the station is situated less than 50 m from the entrance of the major channel where the water residence time is the lowest (20 days) [30]; therefore, the station is mostly influenced by seawater intake rather than inland freshwater. Meteorological data were also collected at a high frequency on the MEDIMEER pontoon less than 5 m from the location of high-frequency water monitoring. The study was carried out from January 7 to May 19, 2015, and from December 1, 2015, to July 6, 2016. Hereafter, these two periods are referred to as 2015 and 2016 for simplicity. No specific permissions were required for the sampling site for the present research activities as the study site is not protected. No endangered or protected species were involved in this work.

### High-frequency monitoring of the meteorological data, Chl *a* fluorescence and physical and chemical properties of the water

For the meteorological parameters (Table 1), air temperature, wind speed and direction, photosynthetically active radiation (PAR, 400–700 nm) and ultraviolet radiation A and B (UVA, 320–400 nm, and UVB, 280–320 nm) were recorded at a high frequency (every 15 min) using a Professional Weather Station (METPAK PRO, Gill instruments) with PAR, UVA and UVB sensors (Skye Instruments).

**Table 1:** Type and acquisition characteristics of the studied variables.

Type of data	Acquisition frequency	Variable	Type of instrument
Meteorological	High frequency: every 15 min	Air temperature	Sensor: Professional Weather Station (METPAK PRO, Gill instruments)
		Wind speed	
		Wind direction	
		PAR (400–700 nm)	Light sensors: Skye Instruments
		UVA (320–400 nm)	
		UVB (280–320 nm)	
Hydrological	High frequency: every 15 min	Water temperature	Sensors: NKE STPS
		Salinity	
		O_2_ concentration	Sensors: AADI Oxygen Optode (Anderaa)
		O_2_ saturation	
		Turbidity	Sensor: ECO FLNTU fluorometer (Wetlabs)
Biological	High frequency: every 15 min	Chl *a* fluorescence	Sensor: ECO FLNTU fluorometer (Wetlabs)
Biological	Weekly	Chl *a* concentrations	Water sample collected by a Niskin bottle and analyzed using high performance liquid chromatography (Waters)
		Phytoplankton abundances (cell diameter: < 6 μm)	Water sample collected by a Niskin bottle and analyzed using flow cytometry (FACSCalibur, Becton Dickinson)
		Phytoplankton abundances (cell diameter: 6–200 μm)	Water sample collected by a Niskin bottle and analyzed using optical microscopy (Olympus IX-70)
Chemical	Weekly	Nutrient concentrations (NO_3_, NO_2_, PO_4_ and Si(OH)_4_)	Water sample collected by a Niskin bottle and analyzed using an automated colorimeter (Seal Analytical)

PAR: photosynthetically active radiation; UVA and UVB: ultraviolet A and B, respectively; O_2_: dioxygen_;_ Chl *a*: chlorophyll *a*; NO_3_: nitrate; NO_2_: nitrite; PO_4_: phosphate and Si(OH)_4_: silicate.

For the water properties at mid-depth (Table 1), the water temperature and salinity were recorded with an NKE STPS sensor (Table 1). The dissolved O_2_ concentration and saturation were recorded using an optical sensor (AADI Oxygen Optode, Aanderaa). The turbidity and the *in situ* Chl *a* fluorescence were recorded with an ECO FLNTU fluorometer (Wetlabs). Water properties were recorded at the same frequency and over the same periods as the meteorological parameters, except for water temperature and conductivity in 2016, where the monitoring started ten days later (from December 11, 2015, to July 6, 2016). All sensors were calibrated before deployment. The temperature sensor was calibrated from 5 to 25°C in 5°C steps using a reference thermometer and thermostatic bath. The salinity sensor was calibrated at 25°C using seawater standards of 10, 30, 35 and 38 (Linearity Pack, OSIL, UK). The 0 standard was made with ultrapure water (MilliQ). The oxygen sensor was calibrated at 0% and 100% O_2_ saturation using the Winkler method [33] for measuring the O_2_ concentration. The Chl *a* fluorescence sensor was calibrated using several types of phytoplankton cultures at various concentrations, with the concentrations measured by spectrofluorometry. All sensors were cleaned weekly to prevent biofouling, and measurement drift was checked after each measurement campaign using the same methods as those used to calibrate each sensor.

### Weekly monitoring of nutrients, Chl *a* concentrations, phytoplankton abundance and diversity

In addition to high-frequency monitoring, water samples were collected weekly to determine nutrient and Chl *a* concentrations and phytoplankton abundances and diversity (Table 1). Samples were collected using a Niskin bottle 1 m below the surface from January 15 to May 12, 2015, and from January 12 to July 6, 2016, between 09:00 and 10:00 am.

To determine the nutrient concentrations, 50 mL seawater subsamples were taken using acid-precleaned polycarbonate bottles and then filtered through Gelman 0.45 μm filters that had been prewashed three times. Then, 13 mL of the filtrate was stored at -20°C until analysis. Nitrate (NO_3_), nitrite (NO_2_), phosphate (PO_4_) and silicate (Si(OH)_4_) concentrations were measured using an automated colorimeter (Seal Analytical) following standard nutrient analysis methods [34].

To determine Chl *a* concentrations, 1 L subsamples were taken, filtered through glass fiber filters (GF/F Whatman: 0.25 mm, nominal pore size: 0.7 μm), and stored at -80°C until analysis. Pigment concentrations, including Chl *a* concentrations, were measured by high performance liquid chromatography (HPLC, Waters) following the extraction protocol described in Vidussi et al. (2011) [34] and the HPLC method described by Zapata et al. (2000) [35].

Phytoplankton (< 6 μm) abundances were estimated by flow cytometry (FACSCalibur, Becton Dickinson), and phytoplankton (6–200 μm) abundances and diversity, by optical microscopy (Olympus IX-70). For the phytoplankton (< 6 μm) abundances, duplicate 1.8 mL subsamples were taken, fixed with glutaraldehyde following the protocol described in Marie et al. (2001) [36] and then stored at -80°C until analysis. Cyanobacteria (< 1 μm), picoeukaryotes (< 3 μm) and nanoeukaryotes (in this study, 3–6 μm cell diameter; see Results) abundances were estimated using the flow cytometry method described by Pecqueur et al. (2011) [37]. Phytoplankton (6–200 μm) abundance and diversity were estimated by microscopy. Duplicate 125 mL subsamples were taken, fixed in 8% formaldehyde and then settled for 24 h in an Utermöhl chamber. Cells of each identified phytoplankton taxon were counted under an inverted microscope. Phytoplankton were identified to the lowest possible taxonomic level (species or genus) using standard keys for phytoplankton taxonomy [38]. Carbon biomasses of phytoplankton analyzed by flow cytometry were estimated using the conversion factors of 0.21 pgC cell^-1^ for cyanobacteria and 0.22 pgC μm^-3^ for picoeukaryotes and nanoeukaryotes [39]. For microscopic observations, phytoplankton biovolumes were estimated for the most common taxa using the best shape [40], and carbon biomasses were then calculated using the conversion factors [41,42].

### Chl *a* fluorescence correction and bloom identification

Chl *a* fluorescence is commonly used as a proxy for phytoplankton biomass. Chl *a* fluorescence data from the fluorometer were corrected using the weekly measurements of Chl *a* concentrations by HPLC to provide coherent high-frequency Chl *a* fluorescence data [43].

Bloom periods were identified by estimating the net phytoplankton growth rate (Eq 1) using the biomass gain or loss [5,9]. The high-frequency Chl *a* fluorescence data over 24 h were used to calculate the daily mean phytoplankton biomass (C_t_). The daily net growth rate (r_t_) was the difference in phytoplankton biomass between two consecutive days. A negative value indicates a biomass loss, whereas a positive r value indicates a biomass gain.

$$r_{t+1}=C_{t+1}-C_{t}$$ (1)

A bloom was identified as a period 1) that started with at least 2 consecutive days of positive growth rates and 2) where the sum of net growth rates over at least 5 consecutive days was positive. The end of the bloom was the day before 5 consecutive days with negative growth. A 1-day peak of net growth was considered a “sporadic event” and not a bloom. When close successions of 5-day blooms were identified, they were coalesced into “bloom periods” followed by “post-bloom periods” to identify key events. The means of the daily net growth rates were calculated for bloom periods to compare the mean growth rates among the different periods.

### Data analysis

Some of the operations required to maintain the quality of the high-frequency sampling, such as sensor recalibration and cleaning or drift correction, occasionally induced single or multiple missing measurements or outliers, which were removed from the data set. In our study, the fraction of missing values for each variable was generally low, i.e., between 0.06% (water temperature, 2015) and 23.48% (O_2_ saturation, 2016). Only the UVA data in 2016 had a higher rejection rate (61.96%), due to a technical problem. Consequently, UVA data were not included in the data analysis for 2016 but were included in the 2015 analysis. All the other missing high-frequency data were estimated using a moving average in a 480 data point window (5 days) [44].

The daily mean of the high-frequency data except for PAR, UVA and UVB was calculated to remove daily variation patterns; for the three exceptions, the daily cumulative value was calculated. The whole data set (i.e., daily values of biological, meteorological and hydrological data) was kept as a separate set for each study period. The two resulting data sets were divided into separate data sets for each period as bloom periods (winter, early spring and spring), post-bloom periods (post-winter bloom and post-early spring bloom) and a winter latency period. The winter latency period was defined as a period where the daily net growth rate was low, with a mean daily net growth rate close to zero. A post-spring bloom in 2015 and a pre-winter bloom in 2016 were identified. However, these blooms were too short to perform a strong statistical test; therefore, we did not keep them in the analysis of identified periods. These separations between different periods therefore provided therefore 5 data sets for 2015 and 4 data sets for 2016. Then, autoregressive and moving average (ARMA) models were used for each time series in each data set to identify ARMA processes [45] before first-differencing the fitted series to remove stationarity [46]. Principal component analysis (PCA) was used on the fitted and first-differenced time series in the 2015 and 2016 data sets to identify the relationships between environmental variables graphically. Then, Spearman’s rank correlation tests were applied pairwise to highlight significant correlations in the 2015 and 2016 data sets. As Chl *a* may exhibit a delayed response to environmental forcing factors, time-lag correlation tests were performed on the 11 different data sets. Time-lag correlations are based on simple Spearman’s rank correlation tests between two variables repeated with time-shifted data to identify the delayed influence of a variable on the Chl *a* dynamics.

The weekly data (nutrient concentrations and phytoplankton abundances and diversity) were kept as a separate data set for each study period but were not divided into identified periods because the quantity of data would have been insufficient (19 data points for 2015 and 23 data points for 2016). For the high-frequency data, ARMA models were used for each time series in each data set when needed. Paired Wilcoxon signed-rank tests were used to compare the mean values of the phytoplankton abundances and diversity and the nutrient concentrations between the 2015 and 2016 data sets. Sample dates were paired by week number (ISO 8601). For example, the sampling dates of January 8, 2015, and January 12, 2016, both corresponding to the 2^nd^ week of the year, were paired. To identify correlations between weekly data and high-frequency environmental data, daily means of the environmental data for the sampling days were added to the weekly data sets before using ARMA models and first-differencing each time series. Spearman’s rank correlation tests were then performed pairwise on the 2015 and 2016 data sets.

## Results

### Bloom identification based on Chl *a* fluorescence data

**Fig 2:**
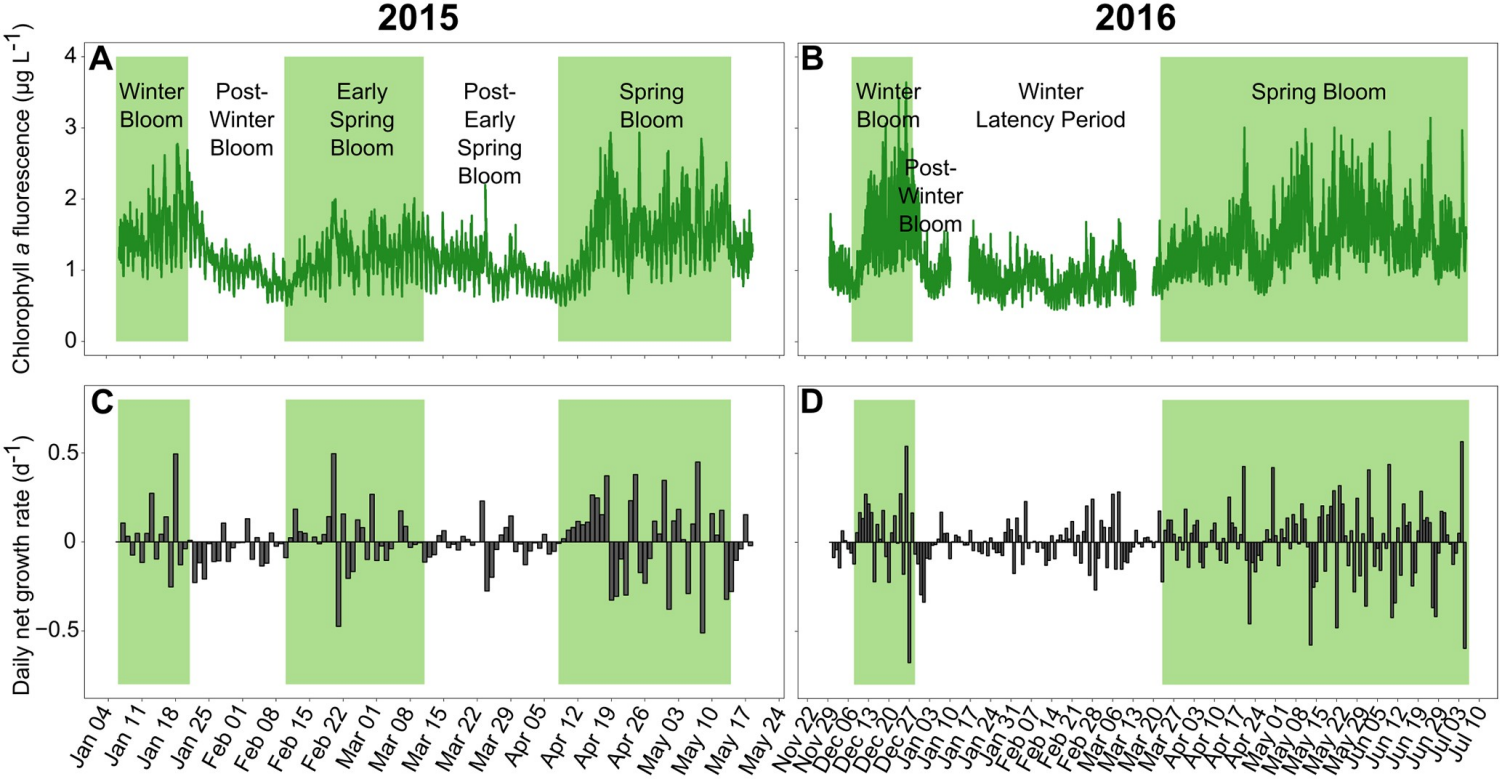
Chlorophyll *a* fluorescence and daily net growth rates. *In situ* Chl *a* fluorescence in 2015 (A) and 2016 (B) and daily net growth rates in 2015 (C) and 2016 (D), indicating daily biomass gains (positive values) and losses (negative values). The bloom periods have a green background, and the post-bloom periods and winter latency period have a white background.

Three blooms were identified in 2015, while only two blooms were identified in 2016 (Fig 2A and 2B). Blooms were defined as consecutive days of biomass gain (positive values of daily net growth rates), while post-bloom periods were consecutive days of biomass loss (negative values; Fig 2C and 2D, respectively). There were winter blooms in January for 2015 and one month earlier, in December 2015, for 2016 (Fig 2A and 2B). The 2016 winter bloom was stronger, with Chl *a* concentrations reaching 3.64 μg L^-1^ in 2016 and 2.77 μg L^-1^ in 2015 (Fig 2A and 2B) and maximum daily net growth rates of 0.54 μg L^-1^ in 2016 and 0.49 μg L^-1^ in 2015 (Fig 2C and 2D). The mean daily net growth rate was 0.055 μg L^-1^ d^-1^ in 2016, almost double the 0.031 μg L^-1^ d^-1^ in 2015. However, it should be noted that the 2015 winter bloom had already started at the beginning of the monitoring. The winter blooms in 2015 and 2016 were followed by post-bloom periods, with the Chl *a* concentration falling to 0.54 μg L^-1^ in 2015 and 0.62 μg L^-1^ in 2016. In 2015, there was an early spring bloom between February 11 and March 11 that was weaker than the preceding winter bloom (maximum Chl *a*: 2.01 μg L^-1^ and mean daily net growth rate: 0.023 μg L^-1^ d^-1^). This early spring bloom was followed by a post-bloom period, with the Chl *a* concentration falling to 0.54 μg L^-1^. In 2016, however, instead of an early spring bloom, there was a winter latency period from January 6 to March 11. During this period, the daily net growth rates were low (Fig 2D), with a mean daily net growth rate close to zero. Then, the main spring blooms occurred from April 9 to May 14 (36 days) in 2015 and from March 24 to July 05 (104 days) in 2016. Notably, the monitoring periods had been planned to end on May 19, 2015, and July 6, 2016; therefore the spring blooms might have continued after these dates. In 2016, the spring bloom showed a maximum Chl *a* concentration of 3.16 μg L^-1^, which was higher than the value of 2.93 μg L^-1^ recorded in 2015, but with a mean daily net growth rate (0.010 μg L^-1^) d^-1^ lower than that (0.022 μg L^-1^) d^-1^ recorded in 2015.

### High-frequency meteorological and hydrological data

The PAR was lowest when the winter blooms occurred in both 2015 and 2016 (Fig 2A and 2B). Then, the PAR slowly increased to reach its maximum of 2688 μmol m^2^ s^-1^ on April 19, 2015, and 2865 μmol m^2^ s^-1^ on June 18, 2016.

**Fig 3:**
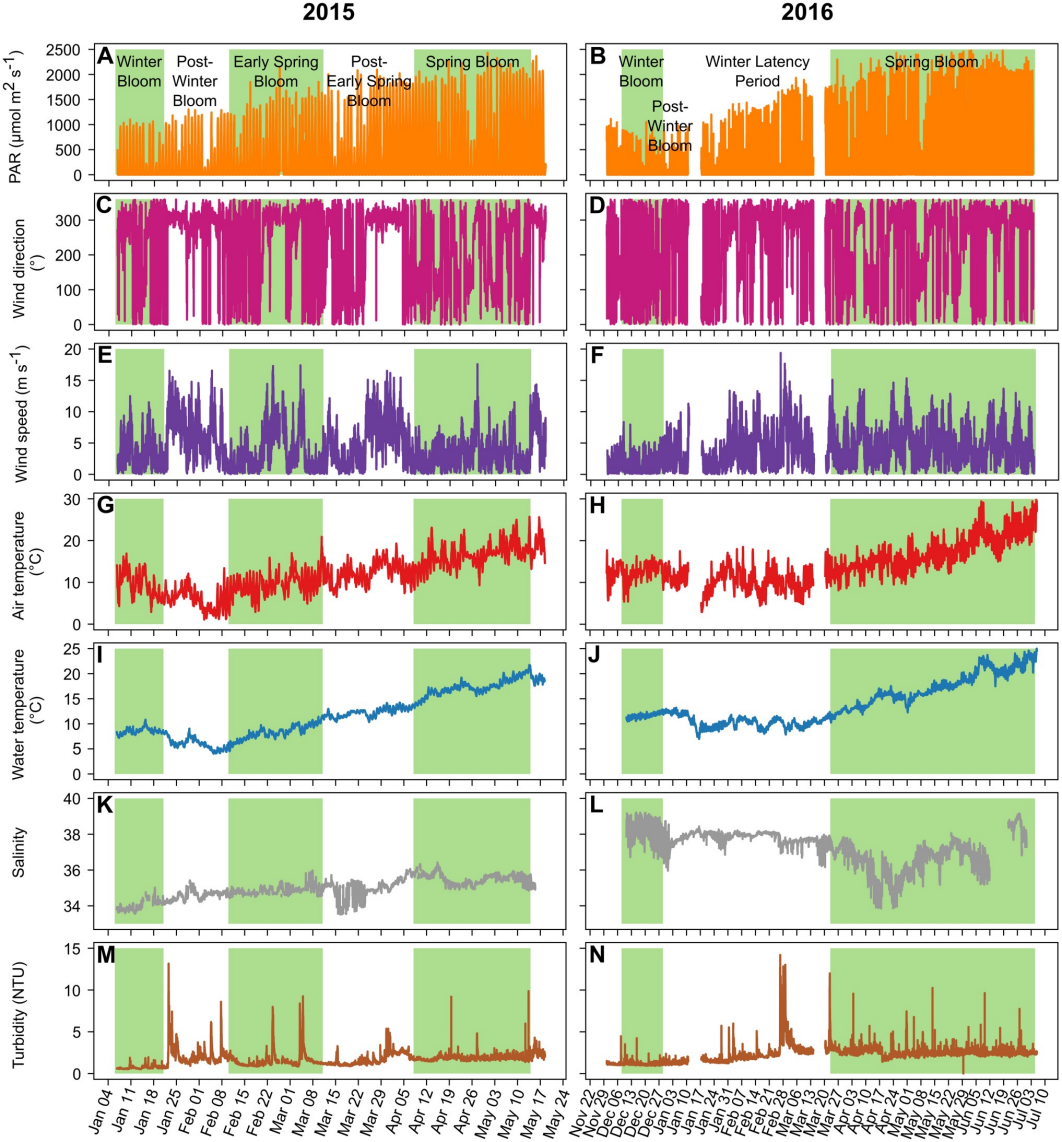
Environmental variables. Main environmental variables for 2015 (left) and 2016 (right). A to H are the meteorological data: PAR (A and B), wind direction (C and D), wind speed (E and F) and air temperature (G and H); and I to N are the hydrological variables: water temperature (I and J), salinity (K and L) and turbidity (M and N). The background colors for the various periods are the same as in Fig 2.

Two dominant winds were identified. Dominance of wind was based on the frequency of occurrence of wind directions over the two studied periods (Fig 3C and 3D). The first wind was from the northwest (49.5% of the data between 270° and 359°), and the second was from the southeast (21% of the data between 90° and 180°). There were three windy periods with northwesterly winds (median of 302°) in 2015 (Fig 3E) during the post-winter bloom period (max = 16.6 m s^-1^), the early spring bloom period (max = 17.4 m s^-1^) and the post-early spring bloom period (max = 16.5 m s^-1^). The wind speed was lower during the winter bloom, the onset of the early spring bloom and the spring bloom (means of 3.3, 1.9 and 3.1 m s^-1^, respectively). In 2016 (Fig 3F), the wind speeds were low during the winter bloom (mean = 1.8 m s^-1^); otherwise, the wind was erratic, with numerous short windy events exhibiting mean wind speed values higher than those observed during the winter bloom.

During the 2015 study period, the mean air temperature dropped from 19.9°C in early January to 1.1°C in early February (Fig 3G). It then increased until the end of the study period, with a maximum of 25.7°C on May 14. During the 2016 study period, the air temperature was stable from early December to mid-March (mean: 10.9±2.7°C, minimum: 2.9°C and maximum: 18.5°C) with a quick chill in early January (Fig 3H). Then the air temperature increased from mid-March until the end of the study period, with a maximum of 30.8°C on July 7.

The water temperature was less variable than the air temperature (Fig 3I and 3J). In 2015, the water temperature was stable during January (8.5±0.6°C), decreased to a minimum of 4.0°C on February 6 and then increased to 21.7°C on May 14. In 2016, the water temperature was 11.8±0.5°C from December 12 to January 11, followed by a quick chill to a minimum of 7.0°C on January 17. Then, the water temperature was stable until March 10 (9.9±0.8°C), followed by an increase until the end of the study period, reaching a maximum of 25.1°C on July 6.

The salinity was also different between 2015 and 2016 (Fig 3K and 3L). In 2015, the salinity was 34.95±0.56, while in 2016, it was higher (37.34±0.95) and more variable and exhibited a large decrease during April, reaching a minimum value of 33.85 on April 18.

The turbidity (Fig 3M and 3N) was 1.69±0.87 NTU in 2015, which was lower than the 2.25±1.06 NTU observed in 2016, with sporadic peaks reaching 13.15 NTU in 2015 and 14.19 NTU in 2016.

### Relationships between Chl *a* fluorescence, meteorological and hydrological data

**Fig 4:**
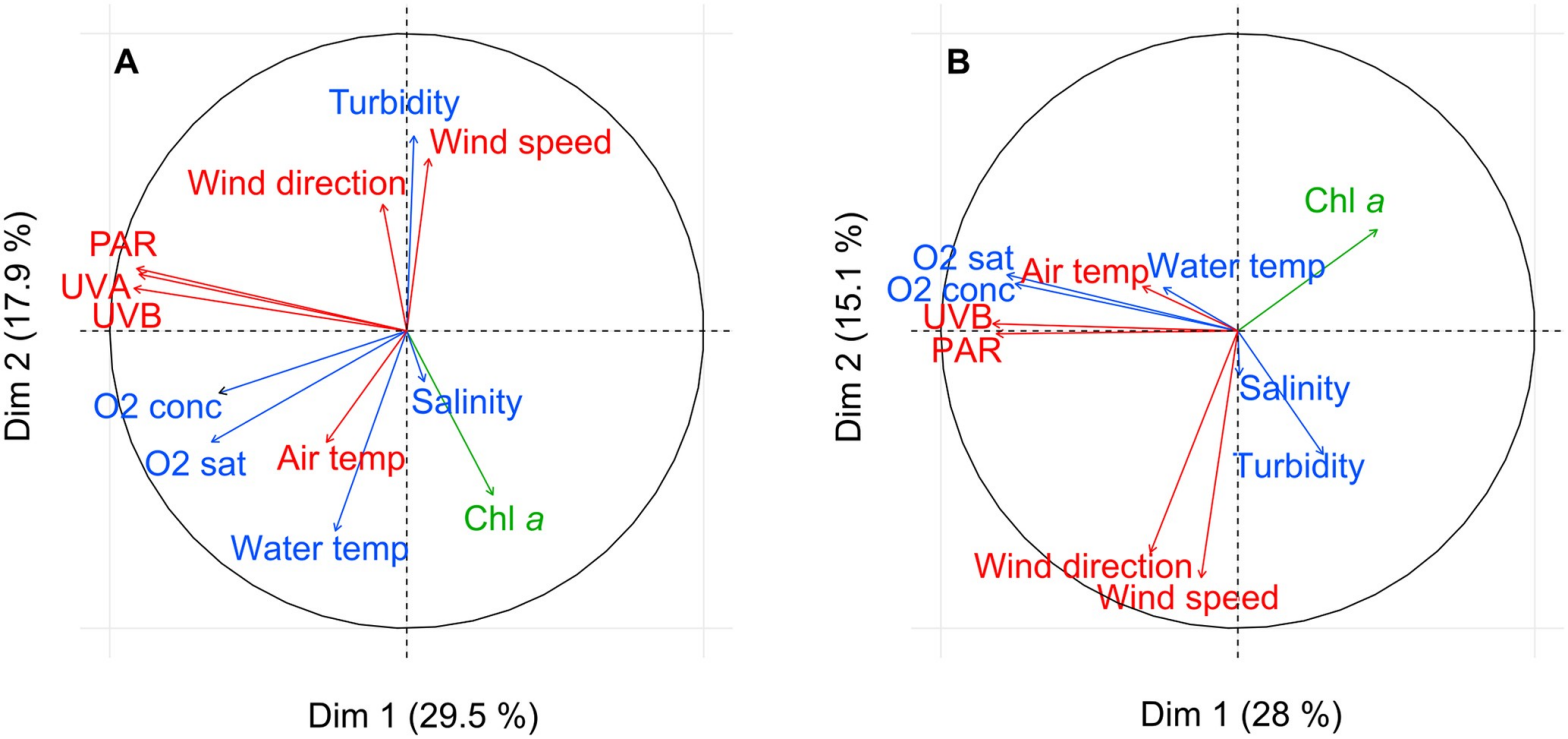
Principal component analysis (PCA) of environmental variables. PCA of Chl *a*, meteorological and hydrological data for the 2015 data set (A) and 2016 data set (B). PCA allows the variables to be projected in multidimensional space to highlight the relationships between them. Here, only two dimensions are represented as they explain the environmental dynamic well. The arrows represent the variables. When arrows are far from the center and close to each other, they are positively correlated, whereas when they are symmetrically opposed, they are negatively correlated. If the arrows are orthogonal, they are not correlated. Finally, when the variables are close to the center, they are not well projected in the dimensions represented; consequently, it is hard to conclude that a relationship occurs between these variables. In this last case, to highlight masked links, we coupled the PCA with pairwise Spearman’s rank correlations as described in the Material and methods.

The general relationships between Chl *a*, meteorological and hydrological data in 2015 and 2016 based on PCA are shown in Fig 4A and 4B. For both data sets, there was a group on the second axis, comprising the wind conditions (speed and direction) and turbidity. Spearman’s rank correlations were strong between wind speed and wind direction for both data sets (2015: ρ = 0.38, *p*-value < 0.001; 2016: ρ = 0.48, *p*-value < 0.001). However, the turbidity was correlated with the wind conditions in 2015 (speed: ρ = 0.41, *p*-value < 0.001; direction: ρ = 0.19, *p*-value < 0.05) but not in 2016. Another group, on the first PCA axis of both data sets, comprised the light parameters, i.e., PAR, UVA (only in 2015) and UVB, and oxygen concentration and saturation. Spearman’s rank correlations were significant between light conditions and oxygen (2015: all *p*-values < 0.01; 2016: all *p*-values < 0.001). Chl *a* fluorescence was opposed to both these groups in both 2015 and 2016, with significant negative correlations with the wind conditions (all *p*-values < 0.05) and the light conditions (all *p*-values < 0.05). The PCA for both data sets also showed a positive correlation between water temperature and air temperature (2015: ρ = 0.36, *p*-value < 0.001; 2016: ρ = 0.29, *p*-value < 0.001). The water temperature was positively correlated with Chl *a* fluorescence in 2015 (ρ = 0.25, *p*-value < 0.01) but not in 2016.

### Time-lag correlations between high-frequency Chl *a* fluorescence, meteorological and hydrological data

As Chl *a* fluorescence may have exhibited a delayed response to environmental forcing factors, the time series were tested for time-lag correlations (Table 2). Chl *a* fluorescence was positively correlated with the water temperature (0- and 5-day lags, strong *p*-values < 0.01) as well as salinity (1- and 3-day lags, *p*-values < 0.05) in both the 2015 and 2016 data sets. As found using PCA, Chl *a* fluorescence was negatively correlated with the light conditions (PAR, UVA and UVB), with a 0-day lags in both 2015 and 2016. However, there was a positive correlation between Chl *a* fluorescence and light conditions with a 1-day lag, but only in 2015. There were negative correlations between Chl *a* fluorescence and wind conditions (speed and direction) with a lag of 0 to 2 days in both 2015 and 2016 (*p*-values < 0.05).

**Table 2:** Time-lag correlations between meteorological and hydrological data.

Year	Period	PAR	UVA	UVB	Wind speed	Wind direction	Air temperature	Water temperature	Salinity	Turbidity
2015	Whole	- - (0;0.27) + + + (1;0.31)	- - (0;0.24) + + + (1;0.32)	- (0;0.22) + + + (1;0.31)	- (0;0.17) - - (1;0.28)	- - (0;0.24)		+ + (0;0.25)	+ (3;0.18)	
	Winter Bloom					+ + (0;0.71) - - (4;0.79)				
	Post-Winter Bloom	- (5;0.56)	- (5;0.55)							+ (3;0.54)
	Early Spring Bloom	- (0;0.40)	- (0;0.39)		+ (5;0.43)			+ + + (0;0.60)	+ + + (0;0.56) - (4;0.43)	- (0;0.38)
	Post-Early Spring Bloom					+ (5;054)				
	Spring Bloom	- (0;0.41) + (1;0.39)	- - (0;0.43) + (1;0.41)	- (0;0.41) + (1;0.39)	+ (2;0.40)	- - (0;0.53)		+ (2;0.35)		
2016	Whole	- - - (0;0.38)		- - - (0;0.36)	- (0;0.17) - (2;0.30)	- - (0;0.30) - (2;0.15)		+ + (5;0.17)	+ (1;0.15) ++ (3;0.21)	+ (0;0.16)
	Winter Bloom					- - (0;0.57)		+ (0;0.54)	- - - (0;0.72)	
	Post-Winter Bloom				- (0;0.86)					
	Winter Latency Period	- (0;0.25) + + (3;0.32)		- - (0;0.31) + + (3;0.34)		- (0;0.24) + + (4;0.32)			+ + (3;0.32)	+ + (0;0.35) - - (1;0.32)
	Spring Bloom	- - - (0;0.44)		- - - (0;0.44)	- - - (0;0.35) + (5;0.21)	- - (0;0.27) - (1;0.22) + (5;0.23)		- (0;0.25) - (2;0.20) + (5;0.21)		

Spearman’s rank time-lag correlations between Chl *a* fluorescence and environmental variables in 2015 and 2016. Whole: tests performed on the whole data set for a study period. Only significant results are shown. The signs + and—represent significant positive and negative correlations, respectively; a single sign represents *p*-value < 0.05; ++ or—represents *p*-value < 0.01; and +++ or—represents *p*-value < 0.001. The time lag (in days) and coefficient of the correlations are in parentheses. For example, +(2;0.40) represents a positive correlation with a *p*-value < 0.05, 2-day lag and coefficient of 0.40. The bloom periods have a green background.

For the separate periods (bloom, post-bloom and winter latency periods), Chl *a* fluorescence was positively correlated with water temperature, with a lag of from 0 to 5 days during four of the five bloom periods (*p*-values from < 0.05 to < 0.001). Chl *a* fluorescence was negatively correlated with the wind conditions (speed and/or direction), with a range of lags between 0 and 4 days for four blooms (*p*-values from < 0.05 to < 0.001). Salinity was positively correlated with Chl *a* fluorescence during the early spring bloom in 2015 (*p*-value < 0.001) and negatively correlated with Chl *a* fluorescence during the winter bloom in 2016 (*p*-value < 0.001). Chl *a* fluorescence was negatively correlated with light conditions for 3 blooms with 0-day lags (*p*-values from < 0.05 to < 0.001), while for the spring bloom in 2015, they were positively correlated with a 1-day lag (*p*-value < 0.05). There was little correlation during the post-bloom periods. Chl *a* fluorescence was negatively correlated with PAR and UVA during the post-winter bloom in 2015 (5-day lags, *p*-value < 0.05) as well as with the wind speed in for 2016 (0-day lag, *p*-value < 0.05) and was positively correlated with the wind direction (5-day lags, *p*-value < 0.05) during the post-early spring bloom period in 2015.

### Nutrient dynamics

**Fig 5:**
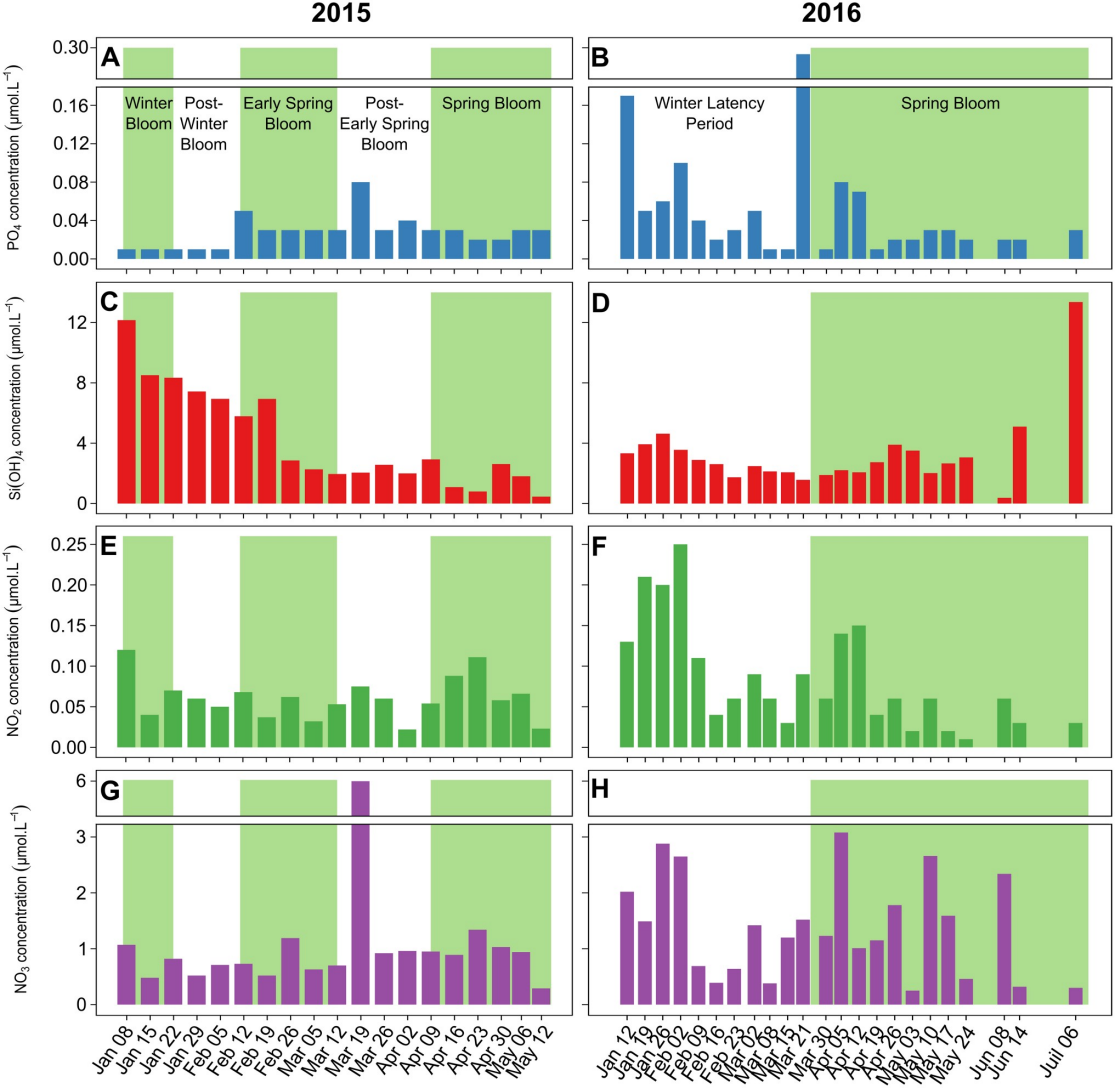
Nutrient concentrations. Nutrient concentrations in 2015 (left) and 2016 (right) for PO_4_ (A and B), Si(OH)_4_ (C and D), NO_2_ (E and F), and NO_3_ (G and H). The background colors for the various periods are the same as in Fig 2.

During the winter bloom and the post-winter bloom periods, the PO_4_, NO_2_ and NO_3_ concentrations were on average 3 to 9 times lower in 2015 (0.01±0.00, 0.07±0.03 and 0.72±0.24 μmol L^-1^, respectively) (Fig 5A, 5E and 5G) than in 2016 (0.09±0.05, 0.20±0.05 and 2.26±0.63 μmol L^-1^, respectively) (Fig 5B, 5F and 5H). The Si(OH)_4_ concentration (Fig 5C and 5D) in 2015 (8.67±2.05 μmol L^-1^) was, however, twice that in 2016 (3.86±0.57 μmol L^-1^) over the same period. In 2015, the Si(OH)_4_ concentrations then gradually decreased until the end of the early spring bloom, reaching a mean of 2.14±0.29 μmol L^-1^ in March.

In 2015, from the early spring bloom to the end of the spring bloom, the PO_4_, NO_2_ and NO_3_ concentrations remained at the same levels as in the winter, even though there were peaks in the PO_4_ (0.08 μmol L^-1^) and NO_3_ (6.01 μmol L^-1^) concentrations on March 12. In 2016, over the same period, the PO_4_, NO_2_ and NO_3_ concentrations fluctuated more than in 2015, but the mean values were similar (0.04±0.06, 0.04±0.02 and 1.24±0.83 μmol L^-1^, respectively). The mean Si(OH)_4_ concentration during the spring bloom in 2015 (1.62±1.00 μmol L^-1^) was approximately half that in 2016 (2.74±0.80 μmol L^-1^).

Wilcoxon signed-rank tests showed that the PO_4_ and NO_3_ concentrations were significantly higher in 2016 than in 2015 (*p*-values < 0.05). The N:P ratios (Redfield ratios) were between 11 and 94 (mean: 45±25) in 2015 and between 6 and 124 (mean: 46±35) in 2016. Spearman’s rank correlations showed significant correlations between Chl *a* and nutrient concentrations in neither 2015 nor 2016.

### Dynamics of phytoplankton abundances

**Fig 6:**
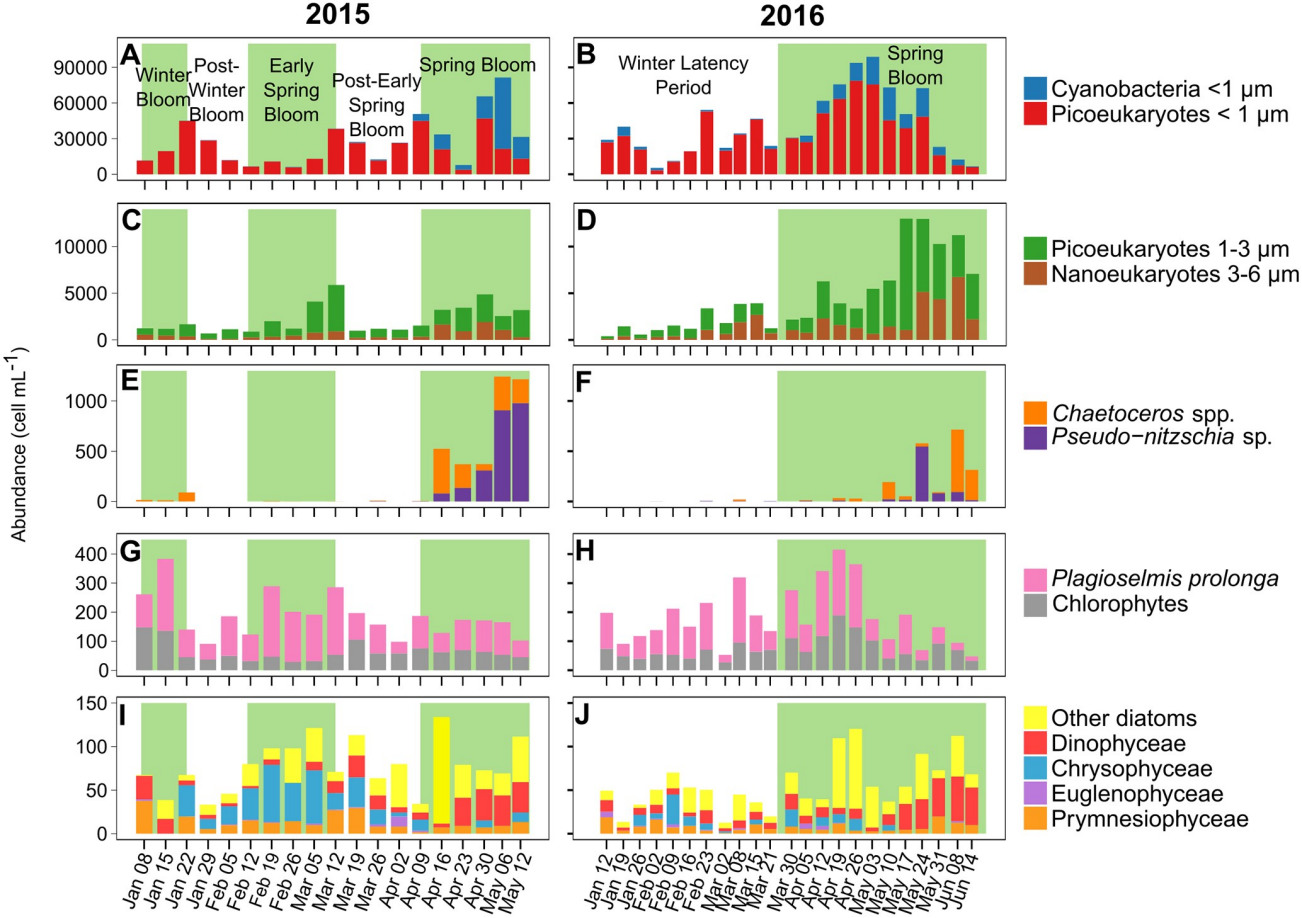
Phytoplankton abundances and diversity. Analyzed by flow cytometry for 2015 (A and C) and 2016 (B and D). Dominant taxa observed by microscopy for 2015 (E, G and I) and 2016 (F, H and J). The background colors for the various periods are the same as in Fig 2.

Three groups of picophytoplankton, namely, cyanobacteria (< 1 μm diameter), picoeukaryotes (< 1 μm diameter) and picoeukaryotes (1–3 μm diameter), and one group of nanoeukaryotes (3–6 μm diameter) were enumerated by flow cytometry (Fig 6A to 6D). The abundances of picoeukaryotes (< 1 μm and 1–3 μm) and nanoeukaryotes were similar between 2015 and 2016: the total abundance of these three groups was 2.14±1.30 × 10^4^ cells mL^-1^ in 2015 and 2.84±1.41 × 10^4^ cells mL^-1^ in 2016. However, the mean abundance of picoeukaryotes and nanoeukaryotes during the spring bloom in 2016 (6.53±1.38 × 10^4^ cells mL^-1^) was more than twice that in 2015 (2.84±1.73 × 10^4^ cells mL^-1^). One of the main differences between 2015 and 2016 was that during winter and early spring, the cyanobacteria (< 1 μm) abundance in 2016 (2.19±2.19 × 10^3^ cells mL^-1^) was almost 10 times higher than that in 2015 (3.02±2.55 × 10^2^ cells mL^-1^). Then, during the spring bloom, cyanobacteria abundances increased to a maximum on May 06, 2015 (6.01 × 10^4^ cells mL^-1^), and a lower maximum on May 10, 2016 (2.70 × 10^4^ cells mL^-1^). The Wilcoxon signed-rank test showed that the mean abundances of cyanobacteria (*p*-value < 0.01), picoeukaryotes (< 1 μm) (*p*-value < 0.01) and nanoeukaryotes (*p*-value < 0.05) were significantly higher in 2016 than in 2015, but there was no significant difference for picoeukaryotes (1–3 μm). Tests for correlations between the biological variables (picophytoplankton and nanophytoplankton abundances and Chl *a* fluorescence) and the environmental variables (wind conditions, light conditions, salinity, air and water temperature, oxygen, turbidity and nutrients) showed a negative correlation of picoeukaryotes (1–3 μm) with PO_4_ concentrations in 2015 (ρ = -0.54, *p*-value < 0.05) and with NO_3_ in 2016 (ρ = -0.50, *p*-value < 0.05). Picoeukaryotes (< 1 μm) were negatively correlated with wind conditions in 2015 (wind speed: ρ = -0.57, *p*-value < 0.05; wind direction: ρ = -0.50, *p*-value < 0.05).

The patterns of community composition and total abundances of phytoplankton (6–200 μm) were different between 2015 and 2016 (Fig 6E to 6J). The total abundances of phytoplankton (6–200 μm) were quite similar during winter and early spring in 2015 (266±81 cells mL^-1^) and 2016 (212±91 cells mL^-1^). During the spring bloom, however, the total phytoplankton (6–200 μm) abundances in 2015 were almost three times those in 2016 (982±433 cells mL^-1^ and 378±123 cells mL^-1^, respectively). This result was confirmed by the Wilcoxon signed-rank test showing that total phytoplankton abundances (6–200 μm) were significantly higher in 2015 than in 2016 (*p*-value < 0.01). The maximum abundances of phytoplankton (6–200 μm) were reached on May 6, 2015, and June 8, 2016 (1470 cells mL^-1^ and 923 cells mL^-1^, respectively). In 2015, during the winter and early spring blooms and their post-bloom periods, the phytoplankton community was dominated numerically by *Plagioselmis prolonga*, a cryptophyte with a diameter of 8–12 μm (41±13%), and chlorophytes (~6 μm) (38.0±8.5%). In addition, chrysophyceae (~6 μm) were also abundant during the early spring bloom (18±2%). In 2016, *P*. *prolonga* dominated the phytoplankton (6–200 μm) community during the winter latency period and the first 11 weeks of the spring bloom (48±11%), and chlorophytes (~6 μm) were also abundant but less abundant than in 2015 (29±9%). *Pseudo-nitzschia* sp. (25–50 μm) and *Chaetoceros* spp. (10–50 μm), which are large, colonial diatoms, successively dominated the phytoplankton (6–200 μm) community during the spring bloom in early April 2015 (72±13%) and the second part of the spring bloom from mid-May 2016 (65±23%) until the end of the study periods. *Chaetoceros* spp. abundances were significantly lower in 2015 than in 2016 (Wilcoxon signed-rank test, *p*-value < 0.05), but *Pseudo-nitzschia* sp. abundances were not.

The phytoplankton community was dominated numerically by picoeukaryotes (< 1 μm), accounting for 51 to 95%, depending on the period (Table 3). However, in terms of carbon biomass, picoeukaryotes were never dominant. In 2015, the carbon biomass was dominated by *P*. *prolonga* in the winter and early spring blooms and by *Chaetoceros* spp. in the spring bloom. Nanoeukaryotes contributed most of the carbon biomass throughout 2016.

**Table 3:** Relative contributions of the dominant phytoplankton groups to the carbon biomass and abundance.

	2015										2016			
	Winter Bloom		Post-Winter Bloom		Early Spring Bloom		Post-Early Spring Bloom		Spring Bloom		Winter Latency Period		Spring Bloom	
	C	Ab	C	Ab	C	Ab	C	Ab	C	Ab	C	Ab	C	Ab
Cyanobacteria (< 1 μm)	0.17	0.62	0.54	0.89	0.38	1.54	1.10	1.92	7.81	40.60	3.48	6.83	5.67	19.82
Picoeukaryotes (< 1 μm)	6.86	**90.50**	16.03	**94.74**	5.49	**79.06**	14.43	**89.49**	2.78	**51.43**	12.38	**86.50**	5.45	**67.86**
Picoeukaryotes (1–3 μm)	4.81	4.07	8.60	3.25	15.05	13.88	16.49	6.54	3.62	4.29	7.96	3.56	9.73	7.75
Nanoeukaryotes (3–6 μm)	28.52	3.01	13.44	0.64	35.26	4.06	**28.87**	1.43	14.26	2.11	**46.23**	2.59	**39.87**	3.97
*Plagioselmis prolonga*	**31.99**	0.92	19.02	0.24	**37.41**	1.17	27.93	0.38	4.50	0.18	22.43	0.34	6.77	0.18
*Chlorophytes*	14.68	0.81	5.61	0.14	4.63	0.28	9.11	0.23	1.58	0.12	5.91	0.17	2.69	0.14
*Chaetoceros* spp.	12.97	0.08	**36.76**	0.10	1.78	0.01	1.92	0.01	**52.96**	0.45	1.43	0.00	27.31	0.17
*Pseudo-nitzschia* sp.	0.00	0.00	0.00	0.00	0.00	0.00	0.14	0.00	12.48	0.82	0.17	0.00	2.50	0.11

Mean relative contribution (in percent) to the carbon biomass (CB) and numerical abundance (Ab) of the dominant phytoplankton groups during each period. Species and period abbreviations and background colors are the same as in Table 2. The highest contribution for each period is in bold.

Correlations between weekly measurements of phytoplankton abundances (6–200 μm), Chl *a* fluorescence and environmental variables were calculated separately for 2015 and 2016. In 2015, *P*. *prolonga* abundance (ρ = -0.60, *p*-value < 0.05) was negatively correlated with PO_4_ concentration, and *Chaetoceros* spp. abundance was positively correlated with Chl *a* fluorescence (ρ = 0.67, *p*-value < 0.01). In 2016, *Pseudo-nitzschia* sp. abundance was positively correlated with Chl *a* fluorescence (ρ = 0.55, *p*-value < 0.05), and *Chaetoceros* sp. abundance was positively correlated with NO_3_ concentration (ρ = 0.58, *p*-value < 0.01).

## Discussion

### Role of water temperature and winter cooling in phytoplankton blooms

Based on the time-lag correlations, water temperature played a significant role in determining Chl *a* dynamics, especially the onset of blooms (Table 2). This is the first time that rising water temperature has been identified as the main factor triggering phytoplankton blooms in an aquatic ecosystem (ocean, coastal zone or lake). This relationship was true for all the main blooms observed in 2015 and 2016, and the strength, number of occurrences and consistent positive sign of the correlations between water temperature and Chl *a* fluorescence suggested that water temperature was the main driver. Other parameters such as wind conditions and salinity were correlated with blooms (see below) but the number of occurrence and non-consistent sign of the correlations suggested that they were not the main drivers. The water temperature was positively correlated with Chl *a* fluorescence with 0 to 5 days of lag suggesting that the biomass accumulation represented by increasing Chl *a* fluorescence was driven by the increase in water temperature over the 5 previous days. Furthermore, every bloom onset corresponded to a period of increase in water temperature, while such correspondence was not detected for other parameters.

The effect of temperature on phytoplankton physiology and metabolic processes is well known. First, under light-saturated conditions, higher temperature increases specific phytoplankton productivity by acting on photosynthetic carbon assimilation [47,48]. In addition, under non-limiting nutrient conditions, an increase in water temperature increases phytoplankton nutrient uptake [49,50]. Moreover, phytoplankton growth rates increases with increasing of temperature, almost doubling with each 10°C increase in temperature (Q_10_ temperature coefficient) [51]. Furthermore, the growth rate of phytoplankton is higher than that of herbivorous grazers at low temperatures [51,52]. Thus, an increase in water temperature, particularly at relatively relative low *in situ* temperatures such as those in this study (6–14°C), can be more favorable for phytoplankton than for their grazers, allowing phytoplankton biomass accumulation, which starts the bloom. Therefore, the initiation of phytoplankton biomass accumulation can result from phytoplankton growth temporarily exceeding grazing-induced losses as a result of increasing temperature.

In previous studies, water temperature was identified as the main driver of blooms of particular species, especially cyanobacteria [53,54]. However, the present study underlined that an increase in water temperature triggered blooms of all phytoplankton community blooms, not just a particular species. Furthermore, the spring blooms started at a temperature of 13.9°C in 2015 and 11.2°C in 2016, while the early spring bloom in 2015 began when the water temperature was 6.1°C. This wide water temperature range for bloom initiations indicates that there is not a threshold water temperature that triggers phytoplankton blooms; instead, blooms are initiated by an increase in water temperature.

Interannual comparison showed that winter 2015/2016 was the warmest winter recorded in France according to Meteofrance (http://www.meteofrance.fr/climat-passe-et-futur/bilans-climatiques/bilan-2016/hiver), which led to exceptionally high winter water temperatures. In contrast in 2015, the winter cooling of the water was typical of this coastal site. As a result, the Chl *a* dynamics in 2016 were completely different from those in 2015 (Fig 1). The absence of an early spring bloom and the slower biomass accumulation during the 2016 spring bloom can be explained by the difference between the meteorological conditions. The abnormally high water temperature during the 2016 winter may have been the cause of biomass stagnation during the winter latency period, slowing phytoplankton biomass accumulation during the spring. The absence of significant winter cooling and the relatively mild water temperatures may have allowed predators (e.g., ciliates and copepods) as well as filter feeders (e.g., oyster and mussels) to remain active during this period and maintain grazing pressure on the phytoplankton [55,56], in turn delaying the ecosystem disturbance required to start a bloom until mid-March, when the water temperature started to rise. Therefore, winter water temperature seems to be a crucial factor influencing the dynamics of the spring bloom in temperate shallow coastal zones. The effect of the winter water temperature on the magnitude of the spring bloom has already been reported [57,58], with larger spring blooms and more phytoplankton biomass after cold winters and smaller spring blooms after mild winters in the Wadden Sea. With global warming, these mild winters could become more frequent [59] and may reduce phytoplankton biomass accumulation during spring blooms. This modification of the bloom phenology might potentially change the structure of the plankton community assemblages and, therefore, directly affect the food web. Such modification is particularly important in shallow temperate coastal zones of economic interest as changes in bloom phenology and magnitude may affect fish and shellfish production.

There were short (2–3 weeks) winter blooms in both 2015 and 2016. Winter blooms are known to occur in marine ecosystems, but they are less common than spring blooms. Winter blooms can be rare, exceptional events [60], but they may occur regularly every year, as in the Bahia Blanca Estuary in Argentina [61] or in tropical and subtropical seas [62–64]. Winter blooms in the coastal site of the present study were recorded once before, in December 1993 [65,66], whereas the present study documented winter blooms of a magnitude similar to that of the spring bloom in two consecutive years. These winter blooms represent an important part of annual primary production (daily mean net growth rates of 0.055 μg L^-1^ d^-1^ in 2015 and 0.031 μg L^-1^ d^-1^ in 2016), providing food for the whole plankton food web, and can sometimes be the most important biomass accumulation event during the year [61]. Winter blooms in shallow coastal zones are generally triggered by a combination of forcing factors such as high nutrient concentrations due to autumn rains, an increase in light penetration into the water column due to a reduction in suspended matter or sediments and low grazing pressure due to low water temperature or tidal conditions [61]. In this study, water temperature was associated with the increase in Chl *a* fluorescence during the winter bloom of 2016. For 2015, however, the winter bloom had already been triggered when the monitoring started, and the link between water temperature and Chl *a* for this winter bloom could not be established as the time-lag correlations could not be determined. Additional observations will be necessary to establish whether an annual winter bloom has become a rule, which might indicate that an ecological shift has occurred in this system. The winter bloom may also have been the result of particular climatic/environmental conditions that occurred during the study period. In fact, even though the impact of the El Niño-Southern Oscillation (ENSO) on the Mediterranean climate is still a source of discussion [67], 2015 was a strong El Niño year with an ENSO Oceanic Niño Index (ONI) value of approximately 2, making it less important than that in 1997–1998 but more than that in 1991–1992 [68], which could potentially explain the exceptional 2015–2016 winter climatic conditions.

### Role of other environmental forcing factors in phytoplankton blooms

Nutrient input from runoff, rain events or sediment resuspension is widely considered a key factor triggering blooms in coastal zones. However, there was no direct link between nutrient concentrations and Chl *a* fluorescence in this study, suggesting that nutrients are not the sole driver of Chl *a* dynamics and that a more complex functioning drives blooms in this mesotrophic system (Fig 5). The absence of a link with nutrient concentrations could be explained by the meteorological conditions of this shallow coastal system, where the wind causes a fairly constant nutrient supply from sediment resuspension, which can maintain the necessary nutrient level for phytoplankton growth but does not produce inputs large enough to reveal show a direct link. The wind conditions (speed and direction) were correlated with Chl *a* fluorescence with 0- to 5-day lags throughout the study periods and during three bloom periods each (Table 2). These correlations may indicate that high-speed winds, generally from the northwest (the tramontane, Figs 3 and 4), reduce biomass accumulation, while low-speed winds, generally from the east and southeast increase biomass accumulation. Millet and Cecchi (1992) [69] already reported this relationship between wind conditions and Chl *a* dynamics in this shallow coastal lagoon. They suggested that a wind speed of 4 m s^-1^ was optimal for balancing the beneficial effect of vertical turbulent diffusion and the detrimental influence of horizontal advection dispersion. In our study, wind speed was significantly correlated with turbidity, which was probably caused by sediment resuspension (in particular, peaks of wind speed coincided with those of turbidity, Fig 3), as has already been reported for shallow coastal zones [65,70,71]. This sediment resuspension, creating a fairly constant nutrient input to the water column, may have maintained phytoplankton production and biomass. With weekly nutrient sampling, the role of nutrient inputs in bloom dynamics may be masked because phytoplankton communities respond quickly to nutrient inputs [72]. The use of high-sensitivity *in situ* nutrient sensors with high acquisition frequencies is crucial for improving our understanding of the effect of nutrient inputs on blooms in such dynamic systems.

There were some correlations between phytoplankton group abundances and nutrient dynamics, especially for PO_4_. Furthermore, the N:P ratio was almost 3 times the Redfield ratio of 16:1, suggesting that PO_4_ could be a limiting factor for some phytoplankton groups during some periods of the year. This result is in agreement with that from a nutrient limitation study of French Mediterranean coastal lagoons [38]. In warmer conditions, as the metabolic demands per unit biomass increase, a higher nutrient supply is needed to support phytoplankton growth. As the nutrient demand increases, stress and competition for nutrients increase and smaller phytoplankton benefit [55,73]. However, the PO_4_ and NO_3_ concentrations were higher in 2016, especially during the winter latency period. This result supports our hypothesis that the predators remain active because of mild water temperatures, in turn maintaining a high grazing pressure and leading to low levels of phytoplankton biomass and thus lower nutrient consumption.

Chl *a* fluorescence and salinity were correlated two times during blooms. Increases in salinity in the studied system can be due to warm and dry periods inducing high evaporation or the inflow of saltier water from the Mediterranean Sea via winds. Otherwise, a decrease in salinity is generally caused by freshwater inputs from rain, runoff or floods. When the salinity variations result from saltier water inflow or from freshwater inputs, nutrients may also be input [37,74]. In this study, correlations between salinity and Chl *a* fluorescence were positive one time (for the early spring bloom in 2015) and negative one time (for the winter bloom in 2016), suggesting that there was an indirect effect on phytoplankton biomass through nutrient inputs rather than a direct physiological effect of salinity [75]. Rain and consequent runoff events may have enriched the system with nutrients, providing a supply for phytoplankton growth. This may have been the case for the onset of the winter bloom in 2016, where a decrease in salinity was observed and corresponded to a 4-day rain event during the initiation of the bloom (https://www.historique-meteo.net). This rain event may have enriched the system with nutrients, facilitating phytoplankton growth. Additionally, saltier water inputs from the sea due to strong winds from the southeast may have led to upwelling of nutrient-rich waters or induced the transport and/or accumulation of phytoplankton in the lagoon by currents. These nutrient enrichments can benefit phytoplankton growth [76,77]; however, nutrients can be rapidly assimilated and thus may have not been detected by our weekly nutrient sampling. Saltier water inputs can also explain the strong positive link detected between salinity and Chl *a* fluorescence during the early spring bloom in 2015. The beginning of this period was characterized by winds coming from the southeast that may have input saltier and more nutrient-rich water from the Mediterranean Sea, in turn contributing to the phytoplankton bloom. The southeasterly wind may also have prevented the dispersion of the accumulated phytoplankton.

One other important result was the lack of correlations between incident light parameters (PAR, UVA and UVB irradiance) and Chl *a* dynamics (Table 2). This lack suggests that in the study system, light conditions are non-limiting for phytoplankton production, at least during winter and spring. The study site is a shallow lagoon in which light reaches a large part of the water column, with a mean attenuation coefficient of 0.35 m^-1^ [78]. In shallow temperate coastal systems, light is often non-limiting, as the light intensities in the water column are generally higher than the saturating light intensities for phytoplankton growth [79]. The non-limiting light was also supported by the occurrence of winter blooms with similar levels of Chl *a* fluorescence as the spring blooms, even though the light intensities were at their lowest level (Figs 2 and 3). Even though light did not appear to have a direct impact on Chl *a* fluorescence in this study, day length may have played a role in bloom timing [80]. There were also some negative correlations between incident light and Chl *a* fluorescence with zero lag, probably due to the inhibition of phytoplankton under high light intensities, as mentioned in the literature [81,82]. Where there were positive correlations between incident light and Chl *a* fluorescence, they exhibited a time lag of 1 to 3 days. One possible explanation for this result is that the phytoplankton responded, after a delay, to the high light conditions by first recovering from light inhibition and then increasing their biomass once acclimatized.

### Small phytoplankton species benefit and diatoms lose out in warmer conditions

The dominant phytoplankton species in terms of carbon biomass in the 2015 winter bloom and the early spring bloom was the cryptophyte *P*. *prolonga* (6–12 μm). *Plagioselmis* is a widespread genus in Mediterranean coastal waters throughout the year and is sometimes considered the key primary producer in these systems [83,84]. *Plagioselmis* and cryptophytes in general “high-quality food” [85], ensuring efficient energy transfer to higher trophic levels. In 2016, there was a winter latency period but no early spring bloom. Nanoeukaryotes (3–6 μm) dominated in terms of carbon biomass during the winter latency period, with higher abundances than those observed in 2015, while the *P*. *prolonga* abundance was the same as that in 2015. Nanoeukaryotes (3–6 μm) and *P*. *prolonga* dominated in terms of biomass and were probably the main sources of available energy for grazers, at least from winter to early spring.

In both 2015 and 2016, the spring bloom was dominated in terms of abundance by chain-forming diatoms. *Chaetoceros* spp. and *Pseudo-nitzschia* sp., dominated the large-phytoplankton community (6–200 μm). Diatoms, including *Chaetoceros* spp. and *Pseudo-nitzschia* sp., are among the most frequent bloom-forming taxa, generally being dominant during spring blooms in coastal zones [17,75]. This is also the case at study sites where spring blooms are usually diatom dominated [86,87]. This dominance is well known and is generally attributed to the fast growth rate of diatoms due to rapid nitrogen uptake (high nitrogen affinity [88]), as confirmed by the correlation found between *Chaetoceros* spp. and NO_3_ concentrations in 2015. This rapid response to nitrogen makes these species more competitive than others during the spring, when conditions are favorable (e.g., nutrients, light and temperature). These large cells with a high fatty acid content are known to be a preferential source food for metazooplankton (e.g., copepods) [89,90], which are consumed by planktivorous fish. However, although *Chaetoceros* spp. dominated the biomass during the 2015 spring bloom, nanophytoplankton (3–6 μm) dominated the 2016 spring bloom. In addition, picophytoplankton (< 1 μm) and nanophytoplankton (3–6 μm) abundances were significantly higher in 2016 than in 2015, while *Chaetoceros* spp. abundances were significantly lower. We suggest that the meteorological conditions and in particular the warm winter of 2016 were probably the causes of these differences. Several experimental studies have also suggested that water warming induces a phytoplankton community shift to picoeukaryote and nanoeukaryote dominance in both fresh and marine waters [26,56,91]. The first hypothesis for the shift in phytoplankton composition between 2015 and 2016 is that the relatively high water temperatures throughout winter and spring in 2016 promoted small phytoplankton (e.g., picophytoplankton) rather than larger ones (e.g., diatoms) due to the higher affinity for nutrients, gas uptake (CO_2_ and O_2_) and maximal growth rate under warmer conditions of the former. This advantage can be explained by the temperature-size relationship, which suggests that smaller organisms are more favored than larger ones in warmer conditions due to faster metabolic processes [56,73]. The second hypothesis is that the warmer winter of 2016 promoted grazers, especially larger ones (e.g., copepods). Heterotrophic protists and metazoans are more sensitive to low temperatures than phytoplankton are, and their grazing activity is higher under warmer conditions. The absence of cooling during the winter may have allowed larger grazers (those that fed on the larger phytoplankton) to remain abundant and active [27,56,92]. Thus, these larger grazers may have reduced larger phytoplankton abundances and thereby made their ecological niche more accessible to smaller phytoplankton. Moreover, large zooplankton feed on small protozooplankton that in turn graze on small phytoplankton, reducing the grazing pressure on small phytoplankton.

According to the first hypothesis, the shift from large phytoplankton to picophytoplankton and nanophytoplankton induced by warming will promote microzooplankton (mostly ciliates), creating an intermediate trophic link between primary producers and copepods. This link will lead to a reduction of the energy transfer from primary production to copepods and in turn to planktivorous fish [26,90]. Warmer water conditions, especially during the winter period, will certainly lead to changes in the plankton community that directly affect the whole food web and ecosystem functioning. In addition, one of the main differences between 2015 and 2016 was cyanobacterial abundance (here, mostly *Synechococcus*). During the winter and the early spring before the bloom, cyanobacterial abundances in 2016 were 10 times higher than those in 2015. Cyanobacteria are known to be strongly controlled by water temperature (and irradiance) and are more abundant during warmer months [75,93,94]. The relatively warm water during the winter and the early spring in 2016, without significant cooling, allowed cyanobacteria to maintain high abundances and compete with other phytoplankton. According to the second hypothesis, the small protozooplankton grazing on cyanobacteria would have been controlled via grazing by larger zooplankton facilitated by warmer conditions, further increasing the population of cyanobacteria. With the general spring water warming in both 2015 and 2016, cyanobacteria became more competitive. Their abundances started to increase when the water temperature was between 12 and 14°C. In some regions, including coastal waters and lakes, cyanobacterial blooms can cause hypoxia and nutrient limitation and can be both environmentally and economically damaging [95,96]. Furthermore, some cyanobacteria can produce toxins that are harmful to most vertebrates, including humans [97], and will cause health concerns if their high abundances become chronic with global warming. Fortunately, this is not the case for the cyanobacteria in our study, which were non-toxic unicellular taxa (e.g., *Synechococcus*).

Picoeukaryotes (< 1 μm) dominated the phytoplankton community in terms of abundance throughout the study, followed by picoeukaryotes (1–3 μm), even though they never dominated in terms of biomass (Fig 6 and Table 3). However, picoeukaryotes are known to have high biomass-specific primary production but are also targeted by microzooplankton grazers (e.g., ciliates), which prevents a major part of daily growth [86]. This relationship suggests that despite the low standing stock of carbon, picoeukaryotes play an important role in transferring carbon to higher trophic levels in coastal zones such as the system studied here [98,99]. As picoeukaryotes became more abundant in the water warming period in 2016, including the spring bloom, it is probable that, with global warming, they and small nanoeukaryotes (3–6 μm), will play a greater role in transferring carbon to higher trophic levels including species of commercial interest.

### Toward a general explanation of bloom initiation in shallow coastal waters and general considerations

The disturbance recovery hypothesis [9,10] suggests that a disturbance factor disrupts the predator-prey interactions that allow phytoplankton growth to outpace grazing losses and thereby creates a bloom. Later, in response to the high phytoplankton biomass, the predator abundance and grazing pressure increase, re-establishing the new predator-prey equilibrium and hence ending the bloom. In the North Atlantic oceanic system, the disturbance factor disrupting predator-prey interactions is the deepening of the mixing layer. In shallow, coastal, non-oligotrophic systems such as Thau Lagoon, there is no deep mixing, and other forcing factors might be the major disturbance triggering phytoplankton blooms.

In these shallow coastal waters, previous studies reported that nutrient inputs via wind (sediment resuspension or water transport) and river inputs mainly drove phytoplankton production [69,75]. However, even if the role of these forcing factors is confirmed by the results presented here, this study highlights for the first time that water temperature seems to be the key factor triggering phytoplankton blooms. An increase in water temperature can stimulate phytoplankton metabolic rates such as carbon assimilation and nutrient uptake [49,50]. As the growth rates of phytoplankton can respond more rapidly than those of grazers [51], phytoplankton growth outpaces the losses by grazing, leading to a net biomass gain and starting the bloom. The diverse sources of nutrient inputs (resuspension by winds, rain or freshwater inputs or seawater intake) with frequent pulses contribute to favorable phytoplankton growth conditions during periods of increasing water temperature. Rising water temperature enhances primary production and leads to biomass accumulation and phytoplankton blooms.

In contrast, there were no clear contemporaneous correlations between the environmental variables, including water temperature, and Chl *a* fluorescence during the post-bloom periods (except with wind speed for the post-winter bloom period in 2016). This lack of correlations suggested that the end of the blooms in these systems is regulated by biological processes, in particular zooplankton grazing activity [12]. After favorable conditions trigger the bloom, the increased phytoplankton biomass allows the predator abundances to increase until the grazing rate exceeds the phytoplankton growth rate, leading to phytoplankton biomass loss during the post-bloom period. This interaction supports the food web transfer of matter in these systems, which are also known to be productive in terms of secondary production.

Moreover, low winter water temperatures are important for conditioning the phytoplankton bloom phenology and composition. If there is no significant winter water cooling, then the blooms are delayed and reduced in magnitude, and the dominant phytoplankton in communities shift to smaller ones (cyanobacteria (< 1 μm), picoeukaryotes (< 1 μm) and nanoeukaryotes (3–6 μm)). Such a shift can affect primary production and the whole food web by reducing the energy transfer to higher trophic levels, promoting small predators over larger ones (microbial food web rather than the classic herbivorous food web [100]). With global warming, mild winters could become increasingly frequent and might potentially, in the midterm, totally change the structure of the plankton communities in the most reactive coastal ecosystems. These changes may propagate to upper trophic levels, especially those including fish and shellfish, and have a major impact on commercially exploited coastal systems such as like the studied site as these productive ecosystems are an essential economic resource for local populations. However, the mild winter effect on blooms and more generally the decadal water temperature increases due to global warming can be different according to the system. In some open or deep-coastal zones, phytoplankton blooms are triggered by upwelling, which provides nutrients from deep nutrient-rich water. As global warming heats more land than it heats ocean surface, it may strengthen alongshore winds favorable to upwelling, potentially increasing nutrient inputs and thus bloom events [101]. In open-ocean systems of low and mid latitudes where blooms are not triggered by upwelling, surface temperature increases due to global warming might intensify ocean stratification, and potentially reduce mixing and thus the nutrient inputs from deep water that promote phytoplankton growth. This reduced nutrient supply could diminish the bloom amplitude, net primary production and phytoplankton biomass [25,28,30] and modify bloom timing [102]. However, at higher latitudes, where incident light is limiting, this stratification increase might improve light conditions favorable to phytoplankton growth in the mixing layer and thus increase net primary production and bloom events [103].

However, in the present study, the monitoring was conducted at only one sampling station in Thau Lagoon. In addition, only the winter and spring of two consecutive years were monitored, and 2015 was a “normal” climatic year while 2016 was an exceptional one with a particularly mild winter. Moreover, it is possible that 2015–2016 was influenced by ENSO or North Atlantic Oscillation (NAO) factors. Finally, the influence of tidal phases (spring/neap) on blooms was not studied as in Thau Lagoon the tidal amplitude is very low and frequently masked by climatic factors such as wind conditions [104]. However, tidal phases can influence Chl *a* fluctuations and blooms in coastal waters with higher tidal amplitude [105].

The crucial challenge concerning coastal waters, one of the most productive marine systems, is how these systems will respond to long-term climatic fluctuations such as those expected with global change. The results and conclusion of the present study are the first evidence of rising water temperature as a main driver of phytoplankton blooms in coastal waters. However, for the reasons mentioned above, to confirm our finding as a general rule, other diverse coastal waters should be investigated with adequate monitoring systems for several consecutive years.

## Supporting information

S1 Data Dataset of the high-frequency biological, meteorological and hydrological parameters and weekly water sampling. Sheets one and two correspond to the high-frequency parameters for 2015 and 2016 respectively; three and four to the nutrients concentrations; five and six to the phytoplankton (< 6 μm) abundances; seven and eight to the phytoplankton (6–200 μm) abundances and diversity; nine and ten to the HPLC Chl *a* concentrations.
(XLSX)
